# Leveraging real-world evidence to personalize adjuvant therapy in HR+/HER2- early breast cancer

**DOI:** 10.1093/jncics/pkaf092

**Published:** 2025-10-26

**Authors:** Yael Bar, Steven J Isakoff, Seth A Wander

**Affiliations:** Tel Aviv Sourasky Medical Center and The Faculty of Medicine, Tel Aviv University, Tel Aviv, Israel; Massachusetts General Hospital Cancer Center and Harvard Medical School, Boston, MA, United States; Massachusetts General Hospital Cancer Center and Harvard Medical School, Boston, MA, United States

The use of gene expression assays (GEAs) to assess the risk of recurrence and guide adjuvant therapy decisions for early-stage hormone receptor–positive (HR+), HER2-negative (HER2-) breast cancer is the current standard of care for this large patient population. In this issue of the Journal, Brufsky and colleagues^1^ evaluate the MammaPrint index (MPI) as a predictor of adjuvant chemotherapy benefit in HR+/HER2- early breast cancer (EBC) using real-world data (RWD) from the prospective, observational FLEX registry.

To demonstrate the prognostic and predictive value of the MPI, two propensity score-matched groups, balanced for clinicopathological factors, were created: one consisted of patients who received adjuvant endocrine therapy alone (ET group) and the other with patients who received adjuvant chemotherapy in addition to endocrine therapy (ET+CT group). Five-year risk of a distant recurrence-free interval (DRFI) event increased significantly with higher MPI risk classification in the ET group (average 5-year DRFI risk of 1%, 3.2%, 10%, and 19.1% for patients classified as Ultralow, Low, High 1, and High 2, respectively), emphasizing the prognostic value of the assay. Additionally, a significant benefit of adjuvant chemotherapy was observed in the MPI High 1 and High 2 risk groups (average improvement in 5-year DRFI risk of 5.6% and 10.9%, respectively), whereas only minimal benefit was recorded for patients in the MPI Low and Ultralow risk groups (average improvement of 1.7% and <1%, respectively), highlighting the predictive value of the assay.^1^

To support clinical interpretation, it is helpful to consider these real-world findings in the context of the MINDACT trial, a prospective, phase 3, randomized, noninferiority study designed to assess whether patients with HR+/HER2- EBC who were clinically high risk but genomically low risk (C-high/G-low) could safely forgo adjuvant chemotherapy if treated with ET alone.^2^ Clinical risk was defined by using the Adjuvant! Online algorithm,^3^ and genomic risk was determined by MPI. The study results were first published in 2016,^2^ with updated findings based on a longer median follow-up of 8.7 years published in 2021.^4^ The initial analysis showed that patients with C-high/G-low HR+/HER2- EBC derived no meaningful benefit from the addition of chemotherapy. Specifically, the 5-year distant metastasis-free survival (DMFS) rate was 94.7% in the ET-alone group compared with 95.9% in the group receiving chemotherapy (hazard ratio [HR] = 0.78, 95% confidence interval [CI] = 0.50 to 1.21), supporting the option to omit chemotherapy in this patient population.^2^ However, with longer follow-up, a small risk reduction associated with chemotherapy was observed, translating into a 2.6% absolute difference in 8-year DMFS (92.0% in the ET plus chemotherapy group vs 89.4% in the ET-alone group; HR = 0.66, 95% CI = 0.48 to 0.92).^4^ Given the timing of the MINDACT study accrual, many patients likely received ET considered suboptimal by today’s standards (eg, without ovarian function suppression [OFS], extended ET, or adjuvant CDK4/6 inhibitors), which may influence current estimates of chemotherapy benefit.

The MINDACT trial was not designed to assess the benefit of adjuvant chemotherapy in patients with genomically high-risk HR+/HER2- EBC, as all patients with both clinically high-risk and genomically high-risk disease who were screened for MINDACT were advised to receive adjuvant chemotherapy. In this regard, the current study based on the FLEX registry provides substantial and clinically relevant evidence beyond that of MINDACT by demonstrating a significant benefit from adjuvant chemotherapy in patients with MPI High-Risk as described above.^1^

Of note, in the MINDACT trial, patients with clinically low-risk tumors who had a high genomic risk (C-low/G-high) had no statistically proven benefit from adjuvant chemotherapy (8-year DMFS rate of 92.3% in the ET plus chemotherapy group vs 90.8% in the ET-alone group (HR = 0.85, 95% CI = 0.53 to 1.37).^4^ Therefore, some physicians were hesitant to order the MammaPrint test for patients with clinically low-risk disease because chemotherapy had not been shown to provide a benefit regardless of the genomic result. This prospective RWD analysis of the FLEX registry provided an opportunity to address that limitation and to specifically examine chemotherapy benefit in the C-low/G-high group. Although the authors note in their discussion that tumors with lower-risk clinical features (eg, T1–T2 or node-negative disease) were well represented within the MPI high-risk group, and therefore suggest that the observed 5-year DRFI benefit with chemotherapy for patients in this group applies to the C-low/G-high subgroup as well, the magnitude of chemotherapy benefit stratified by clinical risk (low vs high) within the MPI high-risk group is not presented.^1^ As a result, it remains challenging to draw firm conclusions regarding this specific subgroup, despite the clear need for such guidance in clinical decision making.

In MINDACT, two important predefined subgroup analyses were made for HR+/HER2- patients within the C-high/G-low group. The first examined outcomes by nodal status, showing that although a modest 2.5% absolute difference in 8-year DMFS was observed among node-negative patients (91.7% in the ET plus chemotherapy group vs 89.2% in the ET-alone group; HR = 0.60, 95% CI = 0.38 to 0.96), no benefit was seen in patients with 1-3 positive lymph nodes (91.2% in the ET plus chemotherapy group vs 89.9% in the ET-alone group; HR = 0.84, 95% CI = 0.51 to 1.37).^4^ These findings are somewhat counterintuitive, because lymph node involvement is traditionally considered a major clinical risk factor for disease recurrence. The current FLEX RWD analysis reinforces these results, showing that positive nodal status did not significantly interact with treatment group in predicting chemotherapy benefit.^1^ These results also align with those of the RxPONDER study, which did not demonstrate a chemotherapy benefit in postmenopausal patients with 1-3 positive lymph nodes and a low or intermediate Oncotype DX score.^5^

The second predefined exploratory analysis in MINDACT assessed outcomes in younger (aged ≤50 years) vs older (aged >50 years) patients, acknowledging that chemotherapy may induce OFS in premenopausal women, which serves as an additional indirect therapeutic effect. Although no chemotherapy benefit was observed in older women, a clinically meaningful absolute difference of 5 percentage points in 8-year DMFS was seen in younger women (93.6% in the ET plus chemotherapy group vs 88.6% in the ET-alone group; HR = 0.54, 95% CI = 0.30 to 0.98).^4^ These results align with findings from the TAILORx^6^ and RxPONDER^5^ studies, which showed that younger women derived benefit from chemotherapy even with lower genomic risk (defined by Oncotype DX score). The current FLEX analysis further suggests that premenopausal status is significantly associated with chemotherapy benefit (HR = 0.08, 95% CI = 0.01 to 0.74; *P* = .025), whereas age alone is not (HR = 0.95, 95% CI = 0.89 to 1.02; *P* = .158).^1^ These findings support the hypothesis that the long-term benefit of adjuvant chemotherapy in premenopausal patients with HR+/HER2- EBC and low genomic risk may be driven primarily by chemotherapy-induced OFS rather than by its direct cytotoxic effects. Future analyses of the FLEX data by age, comparing women in different age groups (eg, <45 vs >50), could further contribute to understanding the mechanism of chemotherapy benefit in premenopausal patients. Additionally, several clinical trials are currently assessing the necessity of adding chemotherapy to ET plus OFS in women with high clinical risk and low or intermediate genomic risk.^7^^,^^8^ Their outcomes will help determine whether the benefit attributed to chemotherapy in previous trials can be achieved with OFS alone.

The landscape of evidence generation in breast cancer research is rapidly evolving, with RWD studies increasingly recognized as a critical complement to randomized controlled trials (RCTs). Although RCTs remain the gold standard for establishing efficacy, they are often constrained by narrow eligibility criteria, limited sample sizes in subgroups, and controlled settings that may not reflect everyday clinical practice. In contrast, RWD studies can capture broader patient populations, longer follow-up periods, and contemporary treatment patterns, offering insights into effectiveness, safety, and utility in real-world settings. However, RWD studies also have inherent limitations, including potential biases from nonrandomized treatment allocation, missing or incomplete data, and variability in data quality across institutions, which can affect the validity of findings. The FLEX registry serves as a compelling example of the value of RWD efforts. By prospectively collecting detailed clinical and genomic information from a large and diverse cohort of patients, FLEX enables robust, practice-informing analyses that are often not feasible with RCTs. Numerous research efforts based on this registry in recent years have helped address key clinical questions across diverse settings and have broadened our understanding of how genomic risk stratification is incorporated into everyday clinical decision making for patients with HR+/HER2- EBC.^9-12^

Although GEAs have traditionally been used to guide decisions related to the addition of adjuvant chemotherapy in HR+/HER2- EBC, a growing body of evidence in recent years suggests that these tools may also inform decision making in a broader range of settings. Several GEAs have been shown to predict the likelihood of achieving pathological complete response to neoadjuvant chemotherapy.^13-15^ In addition, GEAs have demonstrated value in selecting patients who are more likely to benefit from neoadjuvant endocrine therapy.^16^^,^^17^ GEAs are also increasingly used to guide decisions on extended adjuvant endocrine therapy in patients with intermediate or high clinical risk^18-21^ or, conversely, to support the omission of endocrine therapy in those with ultralow risk.^22^^,^^23^ GEAs may also help predict the risk of local recurrence, which could inform radiation therapy decisions.^24^ More recently, an analysis based on the FLEX registry suggested a potential role for MammaPrint testing in guiding the choice of adjuvant chemotherapy regimen. Specifically, patients classified as MPI High 2 appeared to benefit from the addition of anthracyclines, whereas those in the MPI High 1 group did not.^9^ These findings were echoed in an exploratory subanalysis of the TAILORx study, which suggested that only patients with an Oncotype DX Recurrence Score greater than 30 derived benefit from the addition of anthracyclines.^25^ Together, these emerging data highlight a new potential role for GEAs, not only in determining whether chemotherapy is recommended but also in guiding the selection of the most appropriate regimen, with the aim of maximizing efficacy while minimizing unnecessary toxicity.

As the clinical utility of GEAs continues to evolve, integrating insights from both randomized trials and real-world studies will be key to optimizing personalized treatment strategies for patients with HR+/HER2- EBC.

## Data Availability

No new data were generated or analyzed for this editorial.
